# Supplementary figures and images for: Molecular Analysis of Polymyxin Resistance among Carbapenemase-Producing Klebsiella pneumoniae in Colombia
Source: Antibiotics (Basel). 2021 Mar 10;10(3):284. doi: 10.3390/antibiotics10030284 (PMC8035654; doi:10.3390/antibiotics10030284)

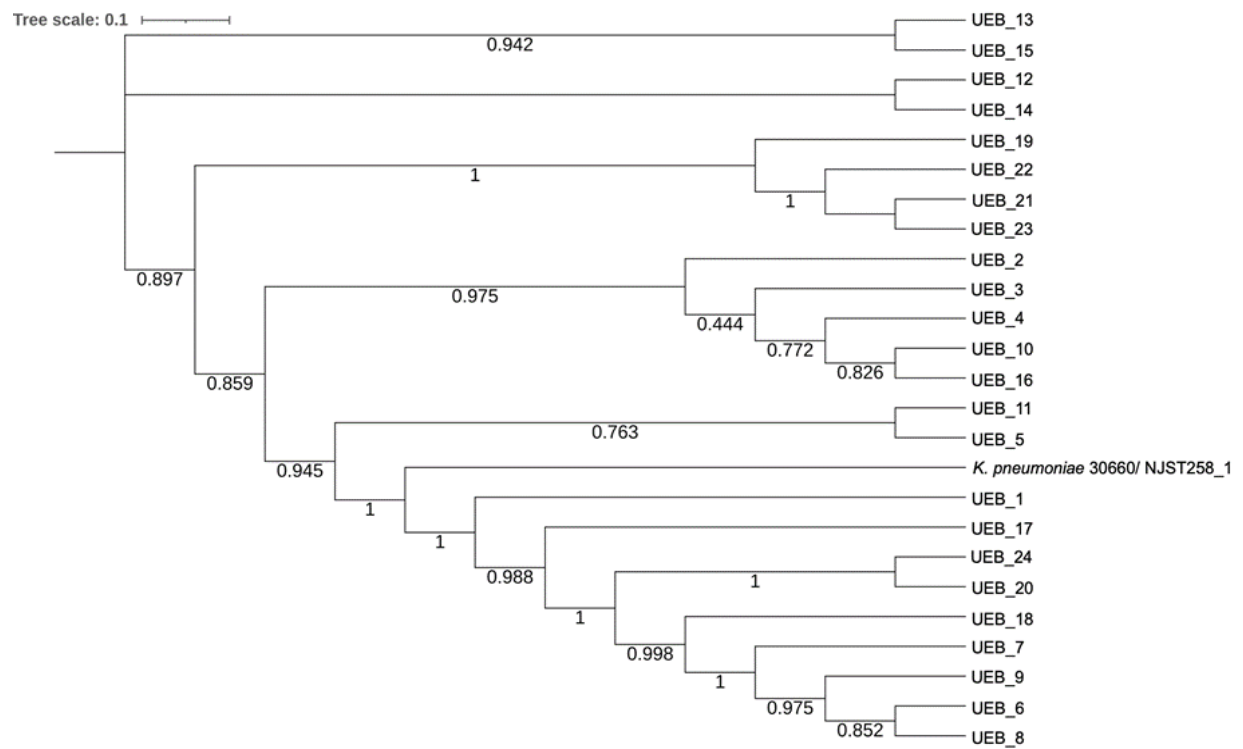

Supplemental Figure S2: Tree inferred by CSI Phylogeny. Bootstrap values shown in each branch.

Supplement: Supplementary file 1 [file antibiotics-10-00284-s001.pdf]
